# Electrochemical Detection of Cancer Biomarkers: From Molecular Sensing to Clinical Translation

**DOI:** 10.3390/bios16010044

**Published:** 2026-01-04

**Authors:** Ahmed Nadeem-Tariq, John Russell Rafanan, Nicole Kang, Sunny Zhang, Hemalatha Kanniyappan, Aftab Merchant

**Affiliations:** 1 Kirk Kerkorian School of Medicine, University of Nevada Las Vegas, 625 Shadow Lane, Las Vegas, NV 89106, USA; nadeemta@unlv.nevada.edu (A.N.-T.); rafanj1@unlv.nevada.edu (J.R.R.);; 2 Department of Biomedical Engineering, University of Massachusetts Amherst, MA 01003, USA

**Keywords:** electrochemical biosensors, cancer biomarker detection, nanomaterials, conducting polymers, healthcare biosensors, graphene and carbon nanotubes, signal transduction

## Abstract

Early cancer detection is crucial for improving survival rates and treatment outcomes. Electrochemical biosensors have emerged as powerful tools for early cancer detection due to their high sensitivity, specificity, and rapid detection capabilities. This review explores recent advancements (2015–2025) in electrochemical biosensors for cancer biomarker detection, their working principles, novel nanomaterial-based enhancements, challenges, and prospects for clinical applications. Specifically, we highlight the electrochemical detection of protein biomarkers (e.g., CEA, PSA, CRP), nucleic acid markers (ctDNA, miRNA, methylation patterns), and metabolic indicators, emphasizing their clinical relevance in early diagnosis and monitoring. Unlike previous reviews which focus on either biomarker classes or sensor platforms, this review uniquely integrates both factors. This review provides a novel perspective on how next-generation electrochemical biosensors can bridge the gap between laboratory development and real-world cancer diagnostics.

## 1. Introduction

Early detection of cancer is widely recognized as a critical factor in improving patient survival and prognosis. Patients diagnosed at an early stage, when tumors are small and localized, have dramatically better outcomes than those diagnosed after metastasis [1]. For example, the 5-year survival rate for common cancers is approximately 91% for those detected at an early localized stage, versus only about 26% for those detected at a late stage. These survival differences underscore why major public health efforts have focused on cancer screening and early diagnosis. Decades of data from high-impact studies (e.g., *JAMA*, *NEJM*) link improvements in cancer mortality to earlier detection. A recent JAMA Oncology modeling analysis estimated that, since 1975, advances in prevention and screening have accounted for roughly 80% of the reduction in mortality for several major cancers [2]. In this context, molecular biomarkers, such as circulating tumor DNA and RNA, cancer-associated proteins, metabolites, and even whole tumor cells shed into biofluids, have become critical indicators for early cancer detection [3].

Electrochemical biosensors have emerged as a promising platform for cancer biomarker detection owing to their rapid response, high sensitivity, analytical specificity, and cost-effective operation [4]. These devices transduce molecular recognition events (such as antigen–antibody binding or nucleic acid hybridization) into quantifiable electrical signals, enabling fast and portable measurements in minimally invasive “liquid biopsy” samples. Recent technological innovations, particularly the integration of nanomaterials, have further bolstered the performance of electrochemical biosensors for cancer diagnostics. By incorporating nanostructured materials, including carbon-based nanostructures (e.g., graphene and carbon nanotubes), metal and metal oxide nanoparticles, and conducting polymers, sensor interfaces achieve dramatically enhanced sensitivity and ultra-low detection limits [5]. These nanomaterials increase the effective electrode surface area and facilitate electron transfer, allowing a higher density of bioreceptors to be immobilized and amplifying electrochemical readouts [6]. For example, coating electrodes with gold nanoparticles, carbon nanotubes, or other nanostructures can concentrate biomolecular probes at the sensor surface, while nanomaterial labels (such as enzyme-tagged nanoparticles) can further amplify the signal generated by a target biomarker. Such nano-engineered electrochemical biosensors have achieved detection of trace-level cancer markers that would be undetectable by conventional assays, underscoring their promise in early cancer screening [6].

A new focus of the field is to translate these advances from the bench to the bedside. Despite significant advancements, most electrochemical biosensors remain confined to research laboratories, and challenges in specificity, reproducibility, and clinical validation have limited translation into routine clinical diagnostics. Complex biological fluids can introduce nonspecific signals and biofouling, and robust sensor operation in real patient samples is still an active area of research. With continued innovation in nanomaterials and device engineering, as well as close collaboration between scientists, clinicians, and regulatory agencies, electrochemical biomarker sensors are expected to move toward clinical translation. This review aims to provide a comprehensive synthesis of electrochemical biosensing technologies for cancer biomarker detection, highlighting recent nanomaterial-based innovations, molecular recognition strategies, and advanced platforms such as CRISPR-based, AI-integrated, and wearable biosensors. The objective is to evaluate how these advancements bridge the gap between laboratory development and clinical translation for early cancer diagnosis.

### Scope and Methodology

This narrative review synthesizes peer-reviewed literature published between 2020 and 2025, retrieved from PubMed, Scopus, and Web of Science using the keywords electrochemical biosensor, cancer biomarker, and nanomaterials. Priority was given to experimental and review articles with clinical or translational relevance. Reference lists of key publications were screened to identify additional studies. Eligible articles focused on electrochemical biosensor technologies for cancer biomarker detection, reported analytical performance (LOD, linear range, sensitivity), or included translational or clinical validation. Non-English and non-peer-reviewed sources were excluded. The review emphasizes comparative evaluation of biosensor types (amperometric, potentiometric, impedimetric, voltammetric) and biomarker categories (protein, genetic, metabolic), highlighting performance metrics and translational readiness.

Compared to previous reviews that focus on either specific biomarker classes or sensing platforms, this review uniquely integrates recent advances in electrochemical biosensing including nanomaterials, CRISPR systems, wearable devices, and AI-enhanced platforms with clinically relevant biomarkers across protein, genetic, epigenetic, and metabolic categories. It emphasizes translational readiness by evaluating real-world performance metrics, regulatory considerations, and current barriers to clinical adoptions. This combined focus on new technology and clinical applicability offers a timely and comprehensive perspective not addressed in existing literature.

## 2. Principles of Electrochemical Biosensors for Cancer Detection

Electrochemical biosensors are analytical devices that can convert biological interactions into a measurable signal. They have gained prominence due to their high sensitivity, low cost, rapid response time, and compatibility with miniaturization and point-of-care testing [7,8]. These biosensors have the capacity to detect biomarkers and integrate recognition elements to produce quantifiable electrical signals in the presence of a specific analyte. In the context of cancer diagnostics, biosensors are able to detect biomarkers such as tumor-associated antigens, circulating nucleic acids, or other abnormal markers that present during the early stages of cancer at low concentrations. This capability makes electrochemical biosensing technology highly sought after, as current cancer diagnostics techniques such as tissue biopsy with histopathologic evaluation (e.g., core-need or excisional tissue biopsy with ancillary immunohistochemistry, while still the gold standard in the industry, suffer from certain limitations such as invasiveness, high cost, and limited sensitivity in the early stages of diseases [8,9,10].

An electrochemical biosensor functions through a coordinated sequence of events involving three main components: the biorecognition element, the transducer, and the signal processor/output. Detection begins when the biorecognition element (i.e., antibody, enzyme, DNA probe, or aptamer) selectively binds to its target biomarker, initiating the sensing event. This molecular interaction is then translated by the transducer into an electrochemical signal, such as a change in current, potential, or impedance. The signal processor and output system take over by amplifying, filtering, and interpreting the signal, ultimately delivering results in a clear, user-friendly format for diagnosis or monitoring [8,11]. The performance of an electrochemical biosensor is determined by the biochemical event (i.e., redox reactions, ionic fluxes, impedance variations), binding affinity between its biorecognition element and target biomarker, the efficiency with which the transducer converts this binding event into an electrical signal, and the effectiveness of any applied signal amplification strategies [7,8]. Therefore, many technology-centered electrochemical biosensors rely on immobilizing a biorecognition element onto a conductive electrode surface and monitoring signal responses when exposed to an analyte. Biosensors have historically been classified by either their biorecognition element (i.e., enzymatic, antibodies, DNA, or aptamers) or transduction method (i.e., electrochemical [amperometric, potentiometric, impedimetric, or voltammetric], optical, or thermal) [7,8,11]. However, these classifications are being expanded in order to reflect on the advancements and implementation of various functionalities, specificities, and device integrations; therefore, this section will review the various types of electrochemical biosensors and their key operating principles [12,13].

### 2.1. Electrochemical Transduction Methods

Amperometric, potentiometric, impedimetric, and voltammetric techniques are the primary electrochemical transduction methods used in cancer biosensing. Owing to their versatility, scalability, and compatibility with a range of biorecognition elements, many electrochemical biosensor platforms are based around these electrochemical transduction mechanisms.

Amperometric biosensors operate by applying a constant potential and measures the current generated from redox reactions. These types of biosensors are often used in enzyme-labeled antibodies or immunosensor platforms where electroactive products are produced. Amperometric biosensors have commonly been used in sandwich immunoassays to quantify cancer biomarker proteins, such as CEA, CA125, and HER2 [7,14,15]. For instance, Ying Zhuo et al., were able to utilize reagentless amperometric immunosensors to detect CEA [15]. While this method of detection can be limited by the dependency on the stability of the biorecognition element and potential background interference with the detected signal, advancements have been made in improving the sensitivity and expanding its integration into different devices and formats [7,12,16,17].

Potentiometric biosensors have the capacity to detect changes in electrode potential due to ion concentration variations without drawing current. These sensors are more suitable for detecting ionic species and less effective for detecting macromolecules such as DNA or proteins [8]. Because potentiometric biosensors are most effective for detecting ionic species rather than large biomolecules, they are typically applied in ion-selective sensors to detect changes in tumor microenvironments—i.e., changes in pH or ionic strength [17]. Recent advancements in potentiometric biosensors have integrated them into field-effect transistor formats to expand the range of detectable biomarkers for label-free detection of exosomes, miRNAs, and circulating tumor DNA [18,19].

Impedimetric sensors, also known as electrochemical impedance spectroscopy (EIS), measure changes in impedance at the electrode surface that reflect biorecognition events, such as analyte binding. EIS has been heavily focused on due to its capacity to offer a label-free, sensitive method of detecting DNA hybridization and antibody–antigen interactions. This type of detection method has already been used for label-free detection of circulating tumor cells, exosomes, DNA methylation patterns, and tumor and protein biomarkers (i.e., miRNA-21 and HER2) [13,20].

Voltammetric sensors detect the resulting current of an applied potential. The potential can be varied, which allows for detailed redox profiles. There are multiple subtypes of voltammetric sensors that vary based on sensitivity, speed, signal clarity, and complexity. These sensors are particularly impactful in multiplexed assays that aim to detect multiple biomarkers using distinct redox-active labels [7,21].

Other electrochemical transduction mechanisms exist that aim to detect unique parameters and enhance diagnostic capabilities. For instance, electrochemiluminescence sensors detect light that is generated from electrochemically triggered redox reactions at the surface of an electrode. This method of detection offers high sensitivity while also minimizing background noise that can be generated in order to produce a clearer signal [22,23]. However, its requirement of instrumental complexity and higher cost makes it unideal when prioritizing simplicity and accessibility. Table 1 below summarizes key advantages, limitations, clinical practicality between the electrochemical transduction methods.

### 2.2. Biorecognition Elements

As noted previously, biorecognition elements are molecular components that selectively bind to specific target biomarkers. They play a critical role in determining the specificity and performance of electrochemical biosensors that depend on the interaction between the biorecognition element and analyte. Commonly employed biorecognition elements include antibodies, aptamers, DNA probes, enzymes, and molecularly imprinted polymers (MIPs). The following section provides an overview of these recognition elements—primarily antibodies, aptamers, DNA probes, enzymes, and MIPs—and their roles in cancer biosensing applications.

Antibodies are highly specific proteins capable of binding to target protein biomarkers, such as CEA, CA-125, or alpha-fetoprotein. These biorecognition elements are commonly used in immunosensors that employ a sandwich format, where a target analyte binds to a captured antibody immobilized on an electrode surface, followed by an additional binding of a detection antibody labeled with an electroactive or enzymatic tag [7]. These biorecognition elements have high affinity and have been extensively utilized due to their capability for diagnostic use; however, antibodies tend to be sensitive to environmental factors (i.e., temperature and pH) that can reduce reliability [7,24].

Aptamers are synthetic single-stranded DNA or RNA oligonucleotides that are folded into three-dimensional structures that allow them to bind to a wide variety of analytes (i.e., proteins, peptides and small molecules). Additionally, this biorecognition element is chemically stable to their environment and can be modified with functional groups; therefore, they confer high specificity. However, despite these advantageous properties, aptamers are limited in the in vivo environment and in clinical translation [8,25].

DNA probes are DNA molecules that rely on complementary base pairings of DNA or RNA sequences. These biorecognition elements have commonly been used to detect oncogenic mutations, circulating tumor DNA, and microRNAs (i.e., miRNA-19b and miRNA-20a) [11,26,27]. These probes offer excellent selectivity and are well suited for detecting nucleic acid targets present in early-stage cancers; however, they are still limited by hybridization efficiency and errors due to interference or mismatching [11].

Enzymatic biorecognition elements take advantage of their catalytic activity and are used to generate electroactive species. This can generate a strong signal amplification and is therefore used as the basis of amperometric and voltammetric methods [8]. However, this biorecognition element can be affected by its environment (i.e., temperature and pH) and prone to influence by the other substances in the matrix (matrix effect) [7,24].

MIPs are synthetic polymers with binding sites that match the target molecule. Since MIPs are generated from templates, the binding cavities used to bind analytes mimic the molecular geometry and chemical functionalities of the target, allowing or selective rebinding. This form of biorecognition allows for both greater internal stability and reusability. Due to relying on non-covalent interactions, however, the binding affinity between the target molecule and MIPs is lower. Additionally, MIPs have been limited in their ability to bind larger molecules and have inconsistencies due to variables that range from template leakage to inconsistent binding kinetics [28,29].

### 2.3. Signal Amplification Strategies

Many cancer biomarkers occur at very low concentrations—often in the femtomolar (fM) to picomolar (pM) range—in biological fluids, particularly during early-stages, making their detection challenging for electrochemical biosensors. To address this, electrochemical biosensor development has focused on improving signal amplification to enhance sensitivity, increase signal-to-noise ratio, and boost overall diagnostic performance [8]. Several signal amplification approaches have been explored and can be divided into nanomaterial-based, enzyme-based, nucleic acid-based, and surface-engineering-based strategies [8,28,30]. This section will discuss the various methods explored to improve the detection capabilities of electrochemical biosensors.

Nanomaterials are materials, such as gold nanoparticles (AuNPs), graphene, carbon nanotubes (CNTs), and quantum dots, that can increase the electrode surface area for the target attachment, improve electron transfer kinetics, and allow for high-density recognition elements to be immobilized on sensor surfaces [8,31]. All of these attributes allow nanomaterials to enhance the sensitivity of detecting analytes. For instance, AuNP-based amplification systems were able to detect HER2 biomarkers in the linear dynamic concentration range of 0.1 pg mL^−1^ to 1 ng mL^−1^ [32]. Enzyme labels, such as horseradish peroxidase (HRP) or alkaline phosphatase, are able to generate catalytic signals and produce electroactive species (i.e., H_2_O_2_), amplifying the measured current. Enzyme labels can also be conjugated to secondary antibodies or nanocarriers (i.e., magnetic beads or nanotubes) to further amplify the signal by increasing enzyme-to-target ratio and enzymatic turnover rate [8]. Prostate specific antigen biomarkers have been able to be detected by HRP-labeled antibodies achieving a limit of detection of 5 fg mL^−1^ [24].

Nucleic acid-based amplification intends to exploit enzymatic or hybridization-based reactions to increase the concentration of detectable nucleic acids. These techniques include rolling circle amplification (RCA), loop-mediated isothermal amplification, and strand displacement amplification, each differing in their sensitivity, rate of detection, and cost. Qian et al., were able to use RCA and surface-enhanced Raman spectroscopy-based sensors to detect miRNA-21 and miRNA-155 biomarkers at low detection limits of 0.4 fM and 0.2 fM, respectively [33]. While originally applied for idiopathic fibrosis risk assessment and chemotherapy monitoring, the same miRNAs are also well-established biomarkers in certain cancers (i.e., non-small cell lung cancer and breast cancer), which demonstrate the platform’s potential applicability in cancer diagnostics [34,35].

Surface engineering strategies typically take form in developing methods of immobilization, such as self-assembled monolayers and layer-by-layer assemblies, sensor surface modifications that improve sensor sensitivity and stability. Specifically, these immobilizing strategies can optimize factors such as recognition element orientation, surface density, anti-fouling properties, and analyte binding selectivity [8,36]. Zhang et al. constructed a nanohybrid platform that allowed the detection of CEA at 1.2 pg mL^−1^, which is notably lower than sensors without nanohybrid surface modifications [36]. Additionally, Karunanidhi et al. integrated nanomaterials alongside surface engineering strategies and nanoengineered a graphene-oxide-coated electrochemical biosensor with integrated machine-learning classification to detect pancreatic-cancer biomarkers with higher sensitivity [37].

Further development in improving diagnostic capabilities includes combining electrochemical detection with optical, magnetic, or fluorescence-based labels [8]. These developments can not only improve sensitivity but also allow for multiplex detection capabilities.

### 2.4. Comparison with Conventional Diagnostic Methods

Comparing modality efficiency, electrochemical biosensors generally offer faster and cheaper assays than traditional methods. A typical sandwich ELISA requires several hours and multiple reagent incubations compared to an optimized electrochemical assay which can generate a signal in seconds to minutes. PCR typically runs in 4–8 h and relies on expensive thermal recyclers. Table 2 lays out comparisons for methods, costs, and detection time of different sensing technologies. 

## 3. Cancer Biomarkers and Their Electrochemical Detection

Biomarkers are a critical tool in diagnosing and monitoring specific cancers, providing valuable insight into disease processes and pathology. Abnormal protein levels, gene expression changes, and metabolite accumulation serve as tumor biomarkers aiding in early cancer detection and potentially guiding [38]. Integration of electrochemical biosensors into clinical practice enhances cancer patient outcomes by offering more real-time, non-invasive, rapid, and low-cost diagnosis and monitoring. This section explores the value of biochemical detection in detecting protein biomarkers, genetic and epigenetic changes, and metabolite levels in improving cancer diagnosis. The biomarkers, types, proposed use, and electrochemical detection are presented in Table 3.

A summary of major cancer biomarkers including protein, genetic, and metabolic categories is outlined in the table. Specifically, their proposed use in clinical practice and electrochemical detection strategies are listed. This table emphases how biomarkers contribute to diagnosis and prognosis of cancers.

### 3.1. Protein Biomarkers

Protein biomarkers are specific proteins found in the body that can indicate the nature, type, and characteristic of a cancer. Biomarkers are often detectable in serum before clinical symptoms manifest and may be present in other biological fluids such as urine or saliva, making them valuable for early diagnosis. Particularly, biomarkers serve as early prognostic factors aiding in monitoring tumor progression and recurrence in various [60,61]. Information regarding the tumor, such as invasiveness, metabolism, angiogenesis, inflammation, and immunosuppression, can be obtained and utilized in management [62]. Commonly established protein biomarkers include C-reactive protein (CRP), carcinoembryonic antigen (CEA), and prostate-specific antigen (PSA), which play a pivotal role in the early detection and monitoring of various cancers after treatment. Within the last decade, much effort has been dedicated to validating electrochemical biosensors as a method of detection of protein biomarkers.

Conventional technologies utilized to detect protein biomarkers include fluorescence in situ hybridization (FISH), polymerase chain reaction (PCR), next-generation sequencing (NGS), flow cytometry, gene expression microarray, immunohistochemistry (IHC), enzyme-linked immunosorbent assay (ELISA), and CRISPR [39]. While these traditional methods are well-researched diagnostic modalities, they require multiple steps, longer processing time, higher costs, and are less adaptable as a point-of-care instrument [39]. Electrochemical biosensors have emerged as an alternative tool or a complementary tool to already existing modalities, providing rapid, low-cost, and portable options for protein biomarker detection. This makes electrochemical biosensors an exciting new avenue of research as well as an ideal tool in a point-of-care setting or in a low-resource environment. Among the many protein biomarkers researched, CRP is associated with cancer-related inflammation, making it a unique marker for electrochemical detection methods.

#### 3.1.1. CRP

CRP is an inflammation marker synthesized by the liver, seen in an array of pathological states such as cancers, infections, injury, and autoimmune conditions [43,63]. In relation to cancer, uncontrolled neoplastic growth disrupts and damages the surrounding local tissues, leading to inflammation [64]. Additionally, the presence of abnormal cancer cells is detected by the adaptive and innate immune system, further exacerbating inflammation. During this state of inflammation, acute-phase reactants such as CRP become elevated. Therefore, CRP is a prominent inflammatory marker released in cancer and can be utilized as a detection target using electrochemical sensors.

Specifically, a concentration threshold level of conventional CRP (>10 μg/mL) in the blood could be associated with an increased risk of cancer, with the strongest correlations observed in gastrointestinal and kidney cancers [46,64,65]. A 2022 prospective cohort and random analysis study analyzed 420,964 cancer-free participants reporting a positive association between elevated CRP levels and subsequent cancer risk [40]. The study reported that incidental cancer patients showcased a higher CRP level compared to the group with no cancer. Detection of elevated CRP levels indicates higher mortality or more malignant tumors. With the advent of electrochemical sensors, more prompt and effective detection of CRP may be achieved.

One useful application of electrochemical sensors in detecting CRP is its extremely high sensitivity. While conventional methods such as ELISA can detect biomarkers, it generally cannot reach the low concentrations, which can limit its use in early detection. Biochemical biosensors can achieve ultrasensitive levels such as a peptide-based biosensor detected CRP in the serum of Crohn patients in the range of 0–0.036 μg/mL with a limit of detection of 0.7 ng/mL [41]. This ultrasensitive CRP biosensor may be especially useful in cancer, as rising CRP levels indicate a higher risk and can be utilized as a method of detection before overt symptoms occur. Furthermore, electrochemical sensors uniquely detect CRP rapidly at low levels. One study described an electrochemical aptasensor that detects CRP at a range of 10 pg mL^−1^ to 10 ug mL^−1^ with a limit of detection (LOD) of 0.44 pg mL^−1^ and a readout time of 5 min [42]. Overall, electrochemical biosensors detect CRP with high sensitivity at lower levels, making them suited for early cancer diagnosis.

As mentioned, conventional methods may take longer processing time, require user expertise of the equipment, and consist of bulkier machines. Label-free electrochemical sensors mark a transition to more adaptive point-of-care diagnostics. For example, conventional methods such as ELSA require a labeling step. Labeling is a crucial part of ELISA in which a detectable signal tag is attached to the antibody, antigen, or probe of interest, like CRP. Current methods of detecting CRP with ELISA include labeling, which makes the process more complex, expensive, and time-consuming. Newer methods of detecting CRP bypass this labeling step, expediting results while keeping accuracy. One such label-free technique involves the use of an origami paper-based electrochemical assay, which measures the binding-induced changes [66]. The accuracy of the origami paper-based electrochemical assay in detecting CRP levels in a human certified sample ranged from 98% to 103.9% in three sensors. Another study similarly utilized a disposable paper-based electrochemical assay to detect CRP with a confidence interval of 95% by *t*-test in comparison to certified human CRP samples [67]. With the advent of paper-based electrochemical assay, it marks a transition to bypassing a more time-consuming step, the labeling step, towards a more rapid and inexpensive method of detecting CRP.

However, limitations of CRP in electrochemical biosensors exist. For one, CRP may lack specificity as elevated levels are attributed to a broad differential seen in many inflammatory states such as infection, trauma, and autoimmune diseases. Therefore, a whole clinical picture is important in addition to prognostic tools like electrochemical biosensor results, which can aid in distinguishing whether elevated CRP values are attributed to cancer alone or to other pathologies. Certain electrochemical biosensors methods collectively profile specific biomarkers which aid cancer diagnosis. For example, CRP is used along with other acute-phase reactants, albumin, and lymphocytes to monitor cancers. One technique utilizes the C-reactive protein-albumin-lymphocyte (CALLY) index, which incorporates multiple acute-phase reactants as a method to predict prognosis. The CALLY index showcased better prognostic value compared to CRP alone [68]. In the realm of electrochemical sensors, utilizing biosensors along with the constellation of symptoms and other laboratory markers, and imaging will guide management.

CRP is well-established in the literature as an excellent tumor biomarker. Future direction should aim to validate electrochemical biosensors as a more effective, quicker, and accurate tool. Electrochemical biosensors have taken a step towards that seen with current studies showing high sensitivity. Novel methods seen with paper-based electrochemical assays, which do not require the labeling step, are also a direction towards more rapid and accurate detection.

#### 3.1.2. CEA

CEA is a glycoprotein found on the surface of cells. It is normally found in low levels in the serum, but can be found elevated, especially in gastric, colorectal, breast, ovarian, and lung cancers [69]. Its main use is to monitor the recurrence of colorectal cancer (CRC) postoperatively, with no strong evidence in its utilization in screening and establishing the diagnosis [44]. In a tissue microarray study, CEA was detectable in 65 of the 120 tumor categories, with CEA positivity most common in colorectal adenomas and carcinomas and other gastrointestinal adenocarcinoma [70].

CEA is particularly important because elevated levels contribute to carcinogenesis, metastasis, and treatment resistance [45]. In terms of detection, CEA is normally found in the serum at a level of 2.5 ng/mL in normal tissues, but levels as high as 100 ng/mL may be suspicious of cancer [69]. Mainly, enzyme-free electrochemical immunosensors demonstrate an optimal strategy to screen for CEA levels within clinically relevant levels [1]. Traditional electrochemical methods usually rely on enzymatic activity to start the cyclic reaction; however, enzymes can be altered by temperature and pH and can increase costs. An enzyme-free method utilizes electroactive substances for the redox strategy to achieve signal amplification offering a more cost-effective and reliable strategy for CEA detection. Overall, higher levels of CEA may indicate more malignant or metastatic states. Hence, it is paramount to utilize a novel method that detects CEA more rapidly to improve survival and guide treatment.

A drawback in measuring CEA is that it is not routinely used to screen, but solely to monitor progression. Reasons include a lack of specificity, as CEA can be found in the gastrointestinal regions, including the pancreas, intestines, and liver. Abnormal levels can reflect pathologies in these areas that may not be due to cancer, making CEA non-specific when other diagnoses can obscure results. Additionally, elevated levels occur in smokers with reports of CEA being as high as 5.0 ng/mL; CEA may also be elevated in benign or malignant conditions [69]. Detection of cancer, therefore, involves the constellation of symptoms as well as other diagnostic findings, which guide treatment.

Electrochemical biosensors have steered towards the direction of developing more novel techniques that may be superior to conventional methods. One technique involves an electrochemical biosensor array that measures multiple cancer biomarkers that aid in early cancer detection. This electrochemical biosensor array uniquely measures the levels of CRP, carbohydrate antigen 12, and CEA in serum, with results comparable to conventional methods [71]. Similarly to CRP detection, there exists an electrochemical paper-based detection of CEA. A paper-based detection improves point-of-care testing, enabling early detection of recurrent. A selective dual-signal electrochemical paper-based device was developed as a cost-effective method to detect CEA as well as 4-nitroquinoline-N-oxide (4-NQO), an oxidative cancer product [72]. Their results showed that the paper-based detection of CEA and 4-NQO was accurate and precise when compared with the standard methods performed in a hospital laboratory.

#### 3.1.3. PSA

PSA is a glycoprotein produced by the prostate gland, most notable for serving as a biomarker for prostate cancer. It is one of the most malignant cancers affecting men globally, accounting for 1,276,106 new cases and causing 3.8% of cancer deaths in men in 2018 [47]. Because of its incidence and mortality rate, prompt detection is essential to enhance patient outcomes and survival.

Electrochemical biosensors in detecting PSA do so with high sensitivity. An aptasensor for PSA utilizes a disposable indium tin oxide polyethylene terephthalate film (ITO-PET). This design showed high precision with a LOD of 8.74 fg/mL PSA [48]. Additionally, when detecting PSA in human serum, the aptasensor yielded accurate results with the RSD (relative standard deviation) ranging from 0.61 to 3.95%. Furthermore, in conjunction with highly sensitive detection of PSA, detection can be made more rapid and cost-effective with label-free strategies. One label-free strategy utilizes silicon nanowires and DNA aptamers. This electrochemical biosensor eliminates the need for labeled reagents, improving the rapid detection of prostate cancer [73]. Utilizing highly sensitive electrochemical biosensors to measure PSA levels may be a cost-effective and sensitive tool for screening and monitoring.

However, PSA levels are known to be abnormal in benign prostatic hyperplasia and prostatitis. Strategies for distinguishing prostate cancer from other pathologies include the integration of other key biomarkers such as vascular endothelial growth factor (VEGF), a protein seen in cancers that promotes the growth of blood vessels. A biochemical biosensor shows promise in differentiating prostate cancer from benign prostatic hyperplasia or prostatitis. This biochemical biosensor utilizes a dual-antibody capable of accurately detecting both VEGF and PSA, providing a novel method in improving diagnostic capability [49].

Recent technological advances in biochemical biosensors detect PSA with accuracy and speed. Innovations include biosensors that are highly sensitive to PSA as well as label-free strategies, which reduce costs and provide rapid results. Additionally, utilizing an approach that incorporates multiple biomarkers, such as VEGF, along with PSA, improves diagnostic accuracy.

The current mode of detection for these markers is more invasive, has longer processing times, and can be inaccurate. Integration of newer models of detection that incorporate multiple biomarkers, such as electrochemical detection, can more accurately specify and detect tumors. In addition to standard modalities of detection, such as imaging, biochemical sensors add confirmation, and electrochemical biosensors detect protein biomarkers such as CRP, CEA, and PSA with high accuracy and sensitivity, helping to guide treatment earlier.

### 3.2. Genetic and Epigenetic Biomarkers

Genetic and epigenetic alterations are critical drivers of oncogenesis. Detection of these changes in the genome can be detected via electrochemical methods, providing insight into cancer development. Epigenetic changes, that is, alterations in genes without altering the DNA sequence itself via DNA methylation, histone covalent modification, or non-coding RNA, occur early in oncogenesis, allowing earlier detection of cancers, which aids in guiding treatment [50]. Genetic changes to the sequence itself are also specific to cancer types helping to distinguish subtypes of cancers or determining malignancy. Hence, detecting early expression of certain genes or alterations to the genome can improve and guide treatment. In this section, we will explore the reliability of electrochemical biosensors in detecting oncogene mutations, microRNA, and DNA methylation patterns.

Electrochemical DNA biosensors detect DNA mutations associated with cancers. Innovations involving electrochemical detection of oncogene mutations primarily revolve around designs that are rapid yet highly sensitive. For example, epidermal growth factor receptor (EGFR) is associated with glioblastoma, a primary malignancy of the central nervous system in adults. With the presence of the blood-brain-barrier, there are limited methods for detecting biomarkers that pass the selective barrier that can then be quantitatively measured in the serum. Exosomes are one of the few markers that can cross the barrier and be found in the serum.

One study exploits this and measures the exosomes that contain the gene, EGFR. This study utilizes a label-free approach that is highly sensitive to measurements of exosomes with a limit of detection of 7.83 × 10^3^ particles/μL [51]. Liquid biopsy components found in the serum, which include exosomes as well as circulating tumor DNA, circulating tumor cells, and tumor-educated platelets, contain genetic biomarkers that guide management [52]. For instance, a reagent less EGFR electrochemical capacitive biosensor provides an accurate yet rapid platform for detecting EGFR in non-small cell lung cancer samples [6]. The study results achieved a response time of 3 s and demonstrated usability up to five times, showcasing a novel method that is affordable and reusable. This design demonstrates strong potential as an effective point-of-care diagnostic tool for EGFR cancers. Analyzing these components is valuable because early detection of genetic biomarkers indicates disease progression and guides treatment. Electrochemical DNA biosensors aim to analyze serum-based genetic biomarkers by detecting key mutations with high sensitivity and specificity.

Electrochemical biosensors measure a component of liquid biopsy in human serum, such as circulating tumor DNA. One study developed a cost-effective DNA biosensor detecting the oncogene KRAS with a time to result of 3.5 h, showing promise as a point-of-care test in detecting cancer and monitoring treatment response [53,54]. Additionally, this biosensor utilizes an oligonucleotide probe-modified sensor coupled to a PCR reaction capable of detecting KRAS mutation despite a complex background of human DNA. Many electrochemical biosensors are working towards this avenue of making detection rapid and accurate. One such is the use of a screen-printed gold electrode (Oviedo, Spain), which detects KRAS in the range of 10 fM to 1 microM [74]. The KRAS gene promotes growth and division, and when altered, can lead to unregulated cell growth, commonly seen in colon, lung, and pancreatic cancers. Many electrochemical biosensors aim to improve the speed and accuracy at which genetic biomarkers such as KRAS, TP53, BRAF, HER2, and BRCA are being detected [75,76,77,78].

MicroRNAs (miRNAs) are non-coding RNAs that are often dysregulated in cancer and therefore serve as a method of early cancer detection [79]. Recent innovations with electrochemical biosensors aim to detect miRNA accurately and rapidly. Conventional methods of detecting noncoding RNA, including microRNAs and long noncoding RNA, include microarray, quantitative RT-PCR, Northern blotting, RNA sequencing, and in situ hybridization [55]. Novel methods, such as biosensor platforms that simultaneously detect multiple miRNAs, detect noncoding RNA with high accuracy [80].

A biochip capable of simultaneously detecting up to 20 miRNAs with a result time of 35 min demonstrated rapid detection, cost-effectiveness, and high sensitivity. A test of five miRNA biomarkers related to breast cancer, miR-125, miR-126, miR-191, miR-155, and miR-21, was utilized in this biochip. Utilizing multiple miRNA biomarkers, especially in cases where multiple subtypes exists, helps to classify the tumor type.

DNA methylation is an important epigenetic component in cancer development and progression. Hypermethylation of tumor suppressor genes and demethylation of tumor oncogenes are the two common modifications that lead to uncontrolled growth of cells and eventual cancer [56]. Prompt recognition of these epigenetic DNA methylation alterations, especially through electrochemical detection, is important in diagnosing and preventing the progression of disease.

Abnormal methylation patterns are strongly associated with cancer progression and therefore serve as critical genetic biomarkers for detection. A superparamagnetic bioengineered polyhydroxybutyric acid nanobead-based electrochemical assay detects methylated DNA in a heterogeneous background of complex human plasma containing various methylation levels [57]. In particular, this biosensor detected DNA methylation levels as low as 5% for 10 ng of input DNA, showing promise as an effective assay. More importantly, the disposable SPE-Au electrodes are inexpensive, and the nanobeads could be reusable, contributing to the cost-effectiveness of the unit.

Beyond assessing the DNA methylation directly, other approaches to quantifying DNA methylation involve measuring an enzyme that influences methylation: DNA methyltransferase. For example, an electrochemical biosensor utilizes [Ru(NH_3_)_6_]^3+^ (RuHex) as a signal transducer to evaluate DNA methylation and methyltransferase activity [58]. This sensor works via attachment of a thiolated single-stranded DNA to a gold electrode that is hybridized with complementary DNA. The formation of a double-stranded DNA contains a recognition site for the methyltransferase. When the double-stranded DNA is methylated by methyltransferase, a methylation-sensitive enzyme cleaves the DNA, resulting in a drop in the electrochemical signal, which corresponds to the increased activity of the methyltransferase. This biosensor is unique as it has a high sensitivity for methyltransferase with a limit of detection of 0.18 UmL^−1^ and measures methyltransferase activity.

Another electrochemical biosensor measures DNA methylation, DNA methyltransferase activity, and screens for methyltransferase inhibitors. This electrochemical biosensor utilizes a platform made of silver nanoparticles and carbon nanocubes to enhance detection achieving a detection limit of 0.03 U/mL with a wider range from 0.05 to 120 U/mL [59]. Compared to conventional methods, this platform allows more sensitive detection of subtle epigenetic changes such as methylation patterns which potentially enable earlier diagnosis. Electrochemical biosensors that measure methyltransferase activity provide sensitive detection of epigenetic changes, offering a novel method for assessing DNA methylation in cancers.

### 3.3. Metabolite-Based Detection

Cancer cells undergo metabolic reprogramming, altering metabolite levels in the serum. Particularly, elevated lactate, altered glucose metabolism, and increased reactive oxygen species provide insight regarding tumor aggressiveness. Metabolite-based detection via electrochemical biosensors is emerging as an approach to monitoring the tumor microenvironment. Insights into the metabolite profiles of cancers can then provide cancer risk assessment and determine prognosis. Advancements in electrochemical biosensors have accurately detected alteration in the tumor microenvironment, improving diagnostic capability.

For example, a microfluidic organ-on-chip system was developed to monitor multiple metabolic levels including oxygen, glucose and lactate in cell cultures in patient-derived triple negative breast cancer stem cells [81]. Their results showcase real-time monitoring on metabolic changes while exposed to drugs such as doxorubicin and actinomycin A. This is particularly important because metabolite consumptions and production can change in response to therapy. This electrochemical biosensor provides a method in determining response to treatment, offering a potential system to manage therapeutic response. Additionally, the biochemical sensors enable continuous monitoring over periods longer than a week aiding in more personalized treatment. This is a unique approach as it links tumor metabolite changes to treatment response utilizing an electrochemical biosensor.

Alterations in metabolism are a feature of many cancers offering a mode of early detection. A recent study introduced a dual-sensing system capable of detecting glucose and lactate providing additional data on metabolism [7]. Both glucose and lactate are important metabolites in cancer pathogenesis, as cancer cells upregulate glycolysis, dramatically increasing glucose consumption and lactate. This system detects glucose and lactate with a non-enzymatic sensor with high accuracy and comparable results to commercial assay kits. Similarly, another dual-sensing system analyzes saliva to simultaneously monitor glucose and lactate levels [8]. This system further showcased that sharing a single cell did not interfere with signal results and that cross-talk between electrodes was negligible. Together, these platforms provide insight into tumor metabolism and highlight a clinically relevant method of detecting cancers.

**Figure 1:**
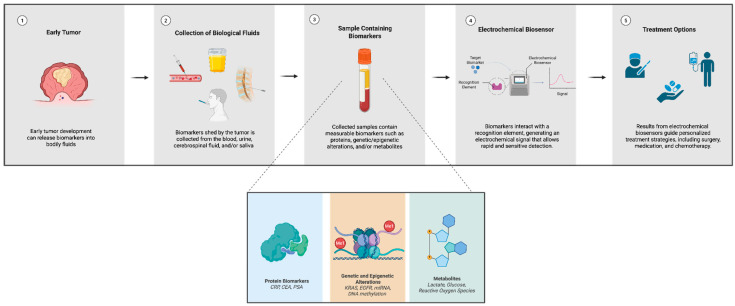
Workflow of Electrochemical Biosensor and Biomarker Detection.

Metabolite-based detection using electrochemical biosensors is a powerful and novel tool for sensing changes in the tumor microenvironment. Cell chips that measure metabolites such as lactate, glucose, and reactive oxygen species offer a non-invasive assessment of cancers. Currently, electrochemical biosensors target cell energy metabolism mechanisms such as glycolysis and mitochondrial pathways which are often dysregulated in oncogenesis [82]. Incorporating devices like the microfluidic organ-on chip platform along with existing methods of detection pave the way for effective and early diagnosis. Figure 1 illustrates the clinical relevance of protein biomarkers, epigenetic and genetic alteration, and metabolite-based detection, and their integration into electrochemical biosensor platforms, which ultimately guides treatment decisions.

This figure illustrates the diagnostic pathway from early tumor development to treatment. (1) Early tumor development can release biomarkers into biological fluids. (2) Biological fluids containing biomarkers are contained in bodily fluids such as the blood, urine, cerebrospinal fluid, or saliva, which can then be collected. (3) Samples containing protein biomarkers (e.g., CRP, CEA, PSA), genetic and epigenetic alteration (e.g., KRAS, EFGR, miRNA, DNA methylation), and metabolites (lactate, glucose, reactive oxygen species) are prepared for testing. (4) Electrochemical biosensors detect target biomarkers that generate signals that are interpreted. (5) Treatment options including surgery, medication, and chemotherapy are guided by the biomarker results.

## 4. Nanomaterials in Electrochemical Cancer Biosensors

### 4.1. Carbon-Based Nanomaterials

Carbon-based nanomaterials such as graphene, carbon nanotubes (CNTs), and carbon nanodots have become helpful in the design of electrochemical biosensors for cancer biomarkers. Their unique physicochemical properties, such as high specific surface area, electrical conductivity, and rich surface chemistry, enable signal amplification and ultra-sensitive detection of molecular cancer indicators [83]. By providing a superior interface between biomolecular recognition elements and electrode transducers, these nanomaterials greatly enhance electron transfer kinetics and loading of capture probes, translating into lower detection limits and improved selectivity in biosensing assays. Importantly, carbon nanomaterial-based electrochemical biosensors are generally biocompatible and can be engineered to be portable and robust, aligning well with the demands of point-of-care cancer diagnostics. In this section, we review the roles of graphene, CNTs, and carbon nanodots in electrochemical cancer biomarker sensing, highlighting their mechanisms for signal amplification, integration with bioreceptors, analytical performance, and examples of translational applications.

#### Graphene

Graphene is a one-atom-thick two-dimensional sheet of sp^2^-hybridized carbon that offers exceptional electrical conductivity and a large, chemically tunable surface for biomolecule immobilization. In electrochemical biosensors, graphene and its derivatives such as graphene oxide, GO; reduced graphene oxide, and rGO have been extensively used to improve analytical performance for detecting a wide range of cancer biomarkers [83]. The atomically flat yet π-rich structure of graphene allows strong π–π stacking and hydrophobic interactions with DNA/RNA strands and aromatic biomolecules, facilitating the stable adsorption or covalent linking of probes such as single-stranded DNA or aptamers [84]. This high probe density, combined with graphene’s excellent electron mobility, leads to rapid electron transfer and amplified currents upon target binding [85].

Graphene-modified electrodes can achieve outstanding sensitivity and low noise in measuring cancer-associated analytes. For example, voltammetric biosensors using graphene have detected oncogenic microRNAs with rapid response and high selectivity, even at sub-picomolar concentrations. In one case, a chitosan-functionalized rGO electrode achieved a detection limit of ~0.28 pM for the protein biomarker VEGFR2 (vascular endothelial growth factor receptor 2), with clear signal enhancement as the target concentration increased in the pM range. Similarly, graphene-based immunosensors have enabled femtogram-level detection of tumor antigens: an electrochemiluminescent graphene oxide/Au nanoparticle platform could quantify the p53 cancer biomarker from 20 to 1000 fg/mL with a limit of detection (LOD) of only 4 fg/mL [86,87]. Such ultra-low LODs (far below pg/mL) are attributed to graphene’s ability to promote electron transfer and to concentrate the antigen–antibody recognition events at the transducer.

**Figure 2:**
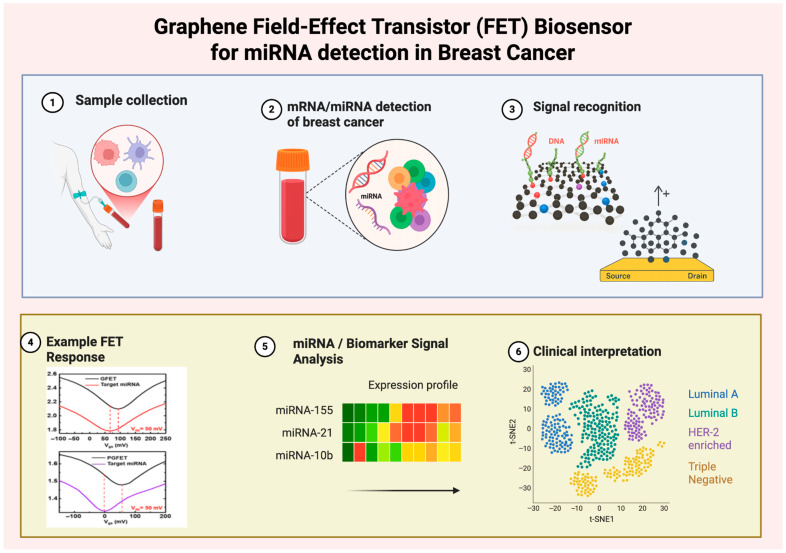
Graphene Field-Effect Transistor (FET) Biosensor for miRNA Detection in Breast Cancer.

Graphene electrodes have been successfully applied to a variety of cancer targets, ranging from protein onco-markers such as PSA, CEA, CA-125, to nucleic acid markers such as tumor suppressor gene mutations, and miRNAs [86]. For instance, graphene-modified electrochemical sensors have detected prostate-specific antigen (PSA) and carcinoembryonic antigen (CEA) at picogram-per-milliliter levels in serum samples. The integration of graphene with magnetic nanoparticles has even enabled the capture and electrochemical detection of whole circulating tumor cells, for example, using magnetic GO conjugated with anti-PSMA antibodies to fish out prostate cancer cells from blood and measure their surface antigen. These examples underscore graphene’s versatile role as a signal-amplifying transducer interface. Graphene-based biosensors are often combined with microfluidics and flexible electronics for translational applications; an illustrative case is a paper-based microfluidic electrochemical device incorporating rGO, which allowed quantitative analysis of cancer biomarkers on-site [88]. Overall, graphene provides an attractive platform for ultrasensitive cancer biosensors, offering a pathway toward early detection with potential in clinical screening and monitoring. Figure 2 and Table 4 help illustrate how graphene field-effect transistors have been used to help detect breast cancer.

A schematic overview of the workflow and signal processing pathway. (1) Blood sample collection containing circulating tumor-derived nucleic acids. (2) Detection of mRNA/miRNA biomarkers associated with breast cancer. (3) Signal recognition via hybridization of nucleic acids on the graphene channel of the FET biosensor. (4) Example FET electrical response curve illustrating shifts in conductance upon target binding. (5) Biomarker signal analysis with expression profiling of relevant breast cancer–associated miRNAs (e.g., miRNA-155, miRNA-21, miRNA-10b). (6) Clinical interpretation of expression profiles enabling molecular subtyping of breast cancer (Luminal A, Luminal B, HER2-enriched, or Triple Negative). Together, this figure highlights the translation of nanoscale molecular interactions on graphene into clinically meaningful breast cancer diagnostics.

### 4.2. Metal and Metal Oxide Nanoparticles

Noble metal nanoparticles such as gold (Au), silver (Ag), and platinum (Pt) have emerged as powerful enhancers in electrochemical cancer biosensors due to their unique electrical conductivity, catalytic activity, and surface bioconjugation capabilities [31]. These nanomaterials exhibit excellent chemical stability and can be functionalized with suitable ligands to confer biocompatibility in biological media [93]. By integrating metal nanoparticles into sensor designs, researchers have achieved improved electron transfer kinetics and a dramatic increase in effective surface area for biomolecule immobilization This has enabled the detection of cancer biomarkers at ultra-low concentrations (far below those detectable by conventional assays) with enhanced sensitivity and lower limits of detection, all while maintaining specificity in complex biological samples. Equally important, Au, Ag, and Pt nanoparticles offer facile surface chemistry for conjugating antibodies, DNA aptamers, and other biorecognition molecules, thereby providing robust platforms for assembling biosensors with high affinity towards target cancer biomarkers.

#### 4.2.1. Gold Nanoparticles (AuNPs)

AuNPs are the most widely used metal nanomaterials in electrochemical biosensors, valued for their high conductivity and strong affinity for thiol- and amine-containing biomolecules. AuNP-modified electrodes can adsorb a dense layer of capture antibodies or DNA probes, effectively increasing the loading of bioreceptors and the surface area available for electrochemical reactions. This translates into higher signal response and lower detection limits [94]. For example, an immunosensor for prostate-specific antigen (PSA) was fabricated by electrodepositing AuNPs onto a glassy carbon electrode, enabling a PSA detection range from 2.0 pg/mL to 10 ng/mL with an impressively low limit of 0.5 pg/mL [95]. Similarly, AuNP-decorated electrodes functionalized with antibodies against carcinoembryonic antigen (CEA) achieved linear detection from 10 fg/mL up to 100 ng/mL of CEA. Such ultralow detection limits (fg–pg/mL) are orders of magnitude beneath typical clinical decision thresholds, underscoring the remarkable sensitivity gains conferred by Au nanostructures. AuNPs can also serve as electrochemical signal labels in sandwich assays, often by conjugation with enzymes or redox reporters to amplify the readout [96]. One strategy is the use of AuNP tags that catalyze a metal deposition reaction: for instance, a PSA immunoassay employed Au nanorod labels followed by silver deposition, and the subsequent anodic stripping of silver yielded a pronounced voltammetric signal for PSA at sub-picogram levels. In another design, a dual aptamer–antibody sensor for the epidermal growth factor receptor (EGFR) utilized AuNPs as a signaling probe, significantly boosting the amperometric response for EGFR detection in the low picomolar range [97]. The versatility of AuNPs is further exemplified by microfluidic immunosensor arrays: by coating microchannel surfaces with AuNP films, multiplexed detection of PSA and interleukin-6 was achieved at sub-pg/mL concentrations in serum. This demonstrates the translational potential of AuNP-enhanced electrochemical sensors to perform sensitive liquid biopsy analyses in complex clinical samples with minimal sample processing. Importantly, AuNP-based biosensors have been successfully validated in real biological matrices (e.g., serum or plasma) without significant signal loss, highlighting their practicality for clinical testing [98].

#### 4.2.2. Silver and Silver Oxide Nanoparticles

**Figure 3:**
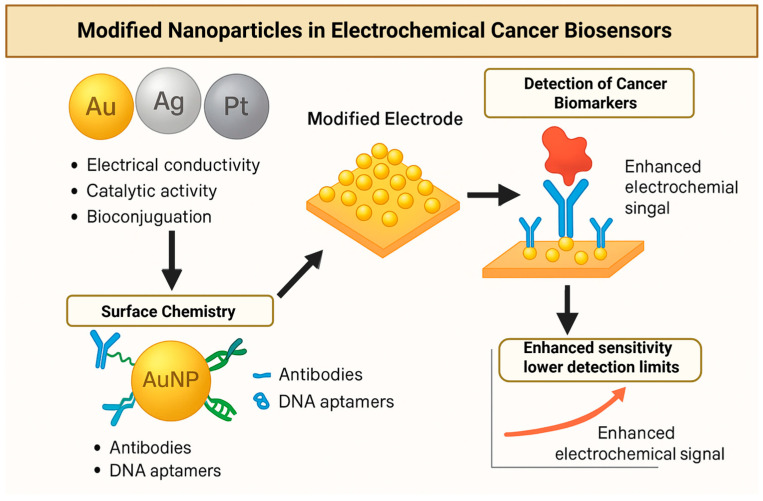
Schematic representation of modified metal nanoparticles in electrochemical cancer biosensors. Gold (Au), silver (Ag), and Platinum (Pt) nanoparticles exhibit high electrical conductivity, catalytic activity, and facile surface chemistry for biomolecule conjugation. Functionalization with antibodies or DNA aptamers increases bioreceptors density on modified electrodes, enhancing electron transfer and signal amplification during target biomarker detection. These properties enable improved sensitivity and lower detection limits in complex clinical samples compared with conventional electrochemical assays. Table 5 shows a summary of various electrochemical cancer biomarker targets.

Ag nanoparticles (AgNPs) offer complementary advantages in electrochemical biosensing, notably extremely high electrical conductivity and efficient electrocatalytic properties for certain redox reactions. While AgNPs can be integrated into electrode nanocomposites (often alongside carbon or graphene materials) to improve electron transfer, they are most prominently used as dynamic signal amplification labels. Silver’s redox activity is readily harnessed in electrochemical readouts—Ag can be oxidized and reduced electrochemically, providing a direct signal, and AgNP labels can catalyze reactions such as the reduction of hydrogen peroxide (H_2_O_2_) to amplify currents [99]. For instance, Ortega et al. developed a voltammetric immunosensor for the epithelial cell adhesion molecule (EpCAM, a CTC marker) using a chitosan film embedded with AgNPs on the electrode; the device detected EpCAM as low as 2.7 pg/mL using an HRP-enzyme secondary antibody to generate an electroactive product [100]. In another study, a graphene oxide electrode coated with hybrid Ag–Au nanoparticles enabled ultrasensitive CEA detection in clinical serum samples, taking advantage of Ag’s catalytic current enhancement and Au’s bioconjugation support [101]. Silver-based labels are also exploited in “metal stripping” assays: Chen et al. reported an anodic stripping voltammetry method in which silver-plated AuNP immunolabels were electro-oxidized, producing a quantitative signal for PSA down to the low femtomolar range [102]. Such approaches leverage each AgNP as a nanoscopic signal source, dramatically amplifying the response (one nanoparticle yields a burst of Ag^+^ ions upon stripping). Moreover, AgNPs can be combined with enzymes or redox dyes for dual amplification; for example, an electrochemical CEA sensor used AgNPs conjugated with glucose oxidase, so that upon binding the target, the Ag catalyzed a redox reaction with enzymatically generated H_2_O_2_, yielding a strong differential pulse voltammetric signal. In summary, silver nanoparticles contribute both as conductive interface enhancers and as potent electrochemical signal tags, enabling detection of cancer biomarkers such as PSA, CEA, and others at exceedingly low concentrations. The inclusion of silver does require careful control bare AgNPs can be prone to oxidation or aggregation, but in stabilized composite forms, they have delivered high sensitivity and even been applied to analyze clinical specimens (e.g., serum) with success. Figure 3 helps demonstrate modified nanoparticles and their use in electrochemical cancer biosensors.

**Table 5:** Summary of Electrochemical Cancer Biomarker Detection Platforms with Metal and Metal Oxide Nanoparticles.

References	Biomarker/Target	Sensor Type/ Modification	Detection Limit (LOD)	Linear Range	Testing Medium	Interference Study	Real-Sample Validation
Huang et al. [101]	Carcinoembryonic antigen (CEA)	Sandwich immunosensor with Ag-Au NPs on graphene	8 pg/mL	10–1.2 × 10^5^ pg/mL	Human serum (spiked)	Not reported	Yes; spiked serum (92–105% recovery)
Ortega et al. [100]	EpCAM (circulating tumor cells)	Microfluidic immunosensor; AgNP chitosan film with HRP tag	2.7 pg/mL	2.7–2000 pg/mL	Spiked human serum/blood	Not reported	Yes; blood serum
Lee et al. [97]	Glucose	GOx/AuNP (AuCL4)/polypyrrole/graphite	Not reported	0.1 pg/mL–0.7 mM	PBS, Human serum	Not reported	PBS, Human serum

### 4.3. Conducting Polymers and Hybrid Nanocomposites

Conducting polymers are an important class of nanostructured materials in biosensors, valued for their electrical conductivity combined with mechanical/chemical versatility. Prototypical examples include polyaniline (PANI) and polypyrrole (PPy), which can be electrosynthesized onto electrode surfaces as thin films or nanostructures. Conducting polymers have been extensively used to improve biosensor performance due to their excellent electrical properties, the flexibility of their surface chemistry, ease of preparation (often by simple electrochemical polymerization), and good biocompatibility [103]. These polymers can effectively interface between biological molecules and electrode surfaces: they are typically porous and can entrap biomolecules during polymerization or be functionalized post-synthesis to attach proteins/DNA, all while providing a conductive matrix that facilitates electron transfer from the biorecognition event to the electrode. For instance, polypyrrole can be electro-polymerized in the presence of an antibody or enzyme, physically entrapping the biomolecule in a conductive PPy film on the electrode [104]. This approach yields a stable, intimately mixed bio-composite where the target binding or enzymatic reaction occurs within a conductive environment, thereby amplifying the electrochemical signal. PANI, on the other hand, is known for its tunable conductivity via doping/dedoping chemistry and has been used in pH-sensing layers and as an intermediate for covalent biofunctionalization. Both PANI and PPy are often grown in nanoscale architectures (e.g., nanofibers, nanowires, nanotubes) to maximize surface area [105]. Notably, conducting polymers tend to be relatively biocompatible—they do not typically denature proteins—and can even reduce non-specific adsorption on electrode surfaces, thus improving the signal-to-background ratio in complex samples.

A major trend in the field is the development of hybrid nanocomposites that combine conducting polymers with other nanomaterials (such as metal nanoparticles or carbon nanostructures) to harness synergistic effects. By integrating two or more components, one can capitalize on multiple mechanisms of signal enhancement simultaneously [106]. For example, a hybrid film of PPy and gold nanoparticles can marry the high loading capacity and conductivity of PPy with the excellent electron transfer and bioconjugation capability of AuNPs. In one study, an electrochemical aptasensor for miR-21 (a microRNA biomarker) was constructed from a nanocomposite of PPy, AuNPs, and graphene on a glassy carbon electrode [107]. The PPy in this composite improved the dispersion and attachment of gold nanoparticles on the electrode, while graphene provided additional conductivity and surface area; together, these components enabled dense immobilization of the DNA aptamer probe and enhanced electron flow [108]. As a result, the sensor could detect miR-21 in a dynamic range from 1.0 fM to 1.0 μM with a remarkably low detection limit (~0.1 fM). Another example of a hybrid is combining *polyaniline, carbon nanotubes, and metal nanoparticles*: a study reported a PANI matrix embedded with multi-walled CNTs and Pt nanoparticles, forming a tri-component nanocomposite on the electrode [109]. This design took advantage of PANI’s conductive polymer network, the added surface area of CNTs, and the catalytic activity of Pt; the resulting biosensor exhibited very high sensitivity and a lower detection limit for a target analyte (in that case, a model analyte for a drug, but the principle translates to biosensors for cancer markers) due to the synergistic catalytic and conductive effects of the composite [110]. Likewise, conducting polymers have been blended with metal oxides (e.g., PANI with Fe_3_O_4_ nanoparticles) and other organic materials to improve stability and sensitivity. One interesting application used a composite of PANI, iron oxide (Fe_3_O_4_), and gelatin to entrap an enzyme for an electrochemical biosensor; the presence of Fe_3_O_4_ provided magnetic controllability and catalytic enhancement, while PANI ensured good electron transport, demonstrating improved analytical performance compared to the enzyme alone on a bare electrode [111].

Importantly, conducting polymer-based platforms often enable the creation of molecularly imprinted polymer (MIP) sensors (as noted in Section 2.2). Polypyrrole and polyaniline can be electropolymerized in the presence of a target analyte or its analog to form imprinted cavities. For example, a molecularly imprinted polypyrrole nanotube sensor was developed for a cancer biomarker and showed excellent specificity and conductivity, benefiting from the nanoscale polymer architecture [112]. Such MIP-based conducting polymer sensors combine the selectivity of the imprinting technique with the signal enhancement of a conductive polymer nanostructure. Another notable advantage of polymer nanocomposites is their flexibility in design: by adjusting the monomer, dopants, or co-materials, one can tailor the polymer’s properties (e.g., hydrophobicity, charge, flexibility) to best suit the target biomarker and sample matrix. As a result, hybrid materials like polyaniline-graphene, polypyrrole-carbon nanotube, or polymer-metal nanoparticle composites are increasingly prevalent in electrochemical cancer biosensors [113]. They have enabled detection of a wide array of cancer biomarkers from protein antigens (using enzyme-loaded polymer matrices for signal amplification) to nucleic acids (using polymer-nanocarbon composites for direct voltammetric readout of DNA binding) often reaching the stringent sensitivity and reliability requirements of clinical diagnostics. The ongoing development of novel conducting polymers and composite materials continues to push the boundaries of biosensor performance, moving electrochemical cancer detection closer to real-world clinical translation.

### 4.4. Nanomaterial-Based Electrochemical Cancer Biosensors for Point-of-Care in Low-Resource Settings

Early cancer detection at the point of care (POC) is important for improving outcomes especially in low-resource settings where there is a lack of advanced laboratory infrastructure. In contrast to conventional lab assays (ELISA or PCR) requiring expensive equipment and skilled technicians, electrochemical devices can be miniaturized and used by primary health workers in the field [114].

Importantly for global health applications, carbon-based biosensors lend themselves to low-cost, portable designs. Carbon electrodes can be created through screen-printing, inkjet printing, or laser scribing, eliminating the need for cleanroom facilities. A recent example is a laser-scribed graphene electrode (Universal Laser Systems, Scottsdale, AZ, USA) embellished with 3D gold nanostructure, which was used in a rapid COVID-19 diagnostic device. This platform is an example of the integration of carbon nanomaterials into a miniaturized electrochemical sensor that could operate at point of care.

When considering metal and metal oxide nanoparticle-based biosensors the quantities of these metals used in nanoparticle-modified sensors are tiny (often picograms to nanograms per sensor), allowing the incremental cost per test to remain low. Additionally, many metal and oxide nanomaterials can be synthesized via wet-chemical methods that are accessible in resource-limited labs. For instance, silver nanoparticles can be “green” synthesized using plant extracts or other benign reagents, and iron oxide nanoparticles can be made by co-precipitation chemistry. A 2024 study demonstrated a green-synthesized graphene/polyaniline nanocomposite sensor for the HER2 breast cancer biomarker, highlighting that an environmentally friendly, low-cost NP production is very feasible [115].

NP labels can enable electrochemical detection without complex instruction, for example, by using anodic stripping voltammetry, where metal labels are detected electrochemically. A case in point is a microfluidic immunosensor for cardiovascular risk biomarker myeloperoxidase (MPO) that combined in situ-grown gold nanoparticles with carbon nanotube electrodes [114]. CNTs acted as a scaffold to electrodeposit gold NPS, which were used to immobilize antibodies for MPO detection. This example illustrates Metal NP-based biosensors have shown promise in POC and near-patient testing due to their reliability and compatibility with miniaturized devices.

## 5. Advanced Strategies for Electrochemical Cancer Biosensors

Emerging technologies are rapidly expanding the capabilities of electrochemical cancer biosensors beyond their traditional applications. Advanced strategies now enable more precise, real-time, and personalized cancer diagnostics through innovations in molecular recognition, data processing, and device integration. CRISPR-based systems offer highly specific genetic detection with single-nucleotide resolution. Wearable and implantable biosensors provide non-invasive, continuous monitoring of tumor-related biomarkers in real-time physiological conditions. Artificial intelligence enhances diagnostic accuracy by uncovering complex patterns in biosensor data, while microfluidic lab-on-a-chip platforms streamline sample handling and multiplex analysis in portable formats. Compared to traditional electrochemical biosensors, these advanced strategies offer greater sensitivity, multiplexing, and real-time monitoring capabilities. However, some challenges remain–such as higher manufacturing costs, biocompatibility concerns, and the need for thorough clinical validation. Thus, future progress likely depends on hybrid approaches that combine the accessibility of traditional biosensors with the precision and intelligence of advanced platforms. Recent developments highlight how these advanced strategies are transforming electrochemical biosensors into powerful tools for early detection, treatment monitoring, and point-of-care cancer diagnostics.

### 5.1. CRISPR-Based Electrochemical Biosensors

The CRISPR-Cas system, originally developed as a gene-editing tool, has been repurposed in biosensing due to its high specificity, programmability, and ability to recognize target nucleic acid sequences with single-base resolution. In recent years, the integration of CRISPR-Cas technologies into electrochemical biosensor platforms has opened new avenues for the detection of gene mutations, oncogenic miRNAs, and circulating tumor DNA (ctDNA) associated with various cancers.

CRISPR-based electrochemical biosensors typically employ Cas12a or Cas13a enzymes, which are guided by a custom-designed RNA to recognize a specific DNA or RNA sequence [116]. Upon recognition, these enzymes exhibit collateral cleavage activity and generate a detectable electrochemical signal. This approach eliminates the need for amplification steps such as PCR, enabling rapid and highly specific nucleic acid detection at room temperature.

A CRISPR/Cas13a-powered microfluidic electrochemical biosensor was developed to detect miRNA-19b and miRNA-20a—both associated with cancer progression—without the need for nucleic acid amplification [27]. The system achieved femtomolar-level sensitivity, indicating strong potential for early-stage cancer diagnostics. Similarly, a CRISPR/dCas9-based biosensing platform for the genotyping of mutant ctDNA, using a specific molecular assembly strategy to enhance electrochemical output [26]. This method enabled high-fidelity mutation detection from complex biological samples, which is critical for precision oncology.

To address single-nucleotide variant detection in patient samples, a Cas12a-based electrochemical sensor was developed for identifying the EGFR L858R point mutation in circulating tumor-DNA from non-small-cell lung cancer. Their platform used Cas12a-mediated trans-cleavage to release methylene-blue-labeled reporters from a nanohybrid-modified electrode, producing a ratiometric current shift. The system reached an ultrasensitive detection limit of 3.3 attomolar and successfully discriminated mutant from wild-type sequences in patient plasma, outperforming droplet digital PCR in sensitivity and accuracy [117].

Further advancing the capabilities of CRISPR biosensing, researchers developed a magnetic nanoparticle–enhanced CRISPR/Cas12a aptasensor for detecting mucin 1 (MUC1), a biomarker overexpressed in breast cancer. The system combines a MUC1-specific aptamer with Cas12a-mediated collateral cleavage to release an electroactive reporter. Magnetic enrichment improved specificity and signal strength, enabling attomolar-level sensitivity and single-base mismatch discrimination [118]. This streamlined, amplification-free platform shows strong potential for clinical and point-of-care breast cancer diagnostics.

Collectively, these CRISPR-based electrochemical biosensors represent a transformative approach to molecular diagnostics, offering amplification-free, highly specific, and ultrasensitive detection of cancer-relevant genetic alterations

### 5.2. Wearable and Implantable Electrochemical Sensors

Recent advances in soft microelectronics, nanomaterials, and wireless telemetry have enabled wearable and implantable devices that continuously track tumor-derived molecules with minute-level resolution. By capturing short-lived fluctuations in circulating analytes, these platforms promise earlier relapse detection, treatment-response feedback, and individualized dosing.

For instance, a flexible electrochemical sensor was engineered using nanoporous ZnO electrodes printed on a polyamide membrane, capable of detecting interleukin-6 (IL-6) in human sweat [119]. Antibodies were stabilized in a room-temperature ionic liquid, allowing for continuous detection at picogram levels with a limit of detection of 0.2 pg/mL and a dynamic range of 0.2–200 pg/mL. Notably, the device retained its sub-picogram sensitivity and selectivity over 24 h of continuous wear without signal degradation, even under mechanical stress from typical user movement.

Meanwhile, in another study, researchers developed a soft, wearable electrochemical biosensor for non-invasive detection of tyrosinase (TYR), a key melanoma biomarker [120]. The device consisted of two formats: a stretchable bandage-style patch and a hollow microneedle platform, both incorporating carbon ink electrodes functionalized with catechol, which served as the electrochemical substrate. In the presence of TYR, catechol is enzymatically oxidized to benzoquinone, generating an amperometric signal measurable in real time. The skin-conformal bandage demonstrated high sensitivity, detecting TYR down to approximately 0.02 µg/mL (20 ng/mL) in gelatin-based phantom models and porcine skin. Additionally, the system integrated a compact (1.9 g) Bluetooth module for wireless data transmission, supporting its potential for on-body melanoma screening without the need for blood sampling.

Advancing this concept further, an adhesive microneedle patch was designed to enable dual-mode detection of tyrosinase (TYR), a melanoma-associated enzyme [121]. The device employed polymer microneedles functionalized with bimetallic Au@Ag-Pt nanostructures, enabling both electrochemical sensing and surface-enhanced Raman spectroscopy (SERS) within the same platform. This configuration allowed for highly sensitive, in situ analysis of intradermal TYR directly beneath the stratum corneum. In a B16F10 melanoma mouse model, the patch successfully monitored dynamic TYR fluctuations over 8 h and achieved an electrochemical limit of detection of 3 ng/mL. Moreover, it could distinguish between malignant and healthy tissue within 5 min, demonstrating its potential for rapid, non-invasive melanoma screening at the point of care.

Furthermore, one study combined microfluidics, printed gold nanodendrite electrodes, and a low-power NFC module for real-time quantification of C-reactive protein (CRP), a key inflammatory and prognostic biomarker in cancer [122]. The patch achieved an ultralow detection limit of 1 pg/mL during sedentary rest and showed strong correlation between sweat and serum CRP levels in patients with heart failure and COPD.

These different platforms showcase how truly minimally invasive electrochemical devices are reshaping cancer surveillance. These first-generation wearables demonstrate that sweat can be mined for cytokines, enzymes, and acute-phase proteins at concentrations relevant to cancer onset and progression, supplying continuous molecular feedback that was previously accessible only through invasive blood draws. Collectively, they affirm that sweat and shallow interstitial fluid can serve as reliable, needle-free windows into tumor biology, enabling continuous, patient-centric monitoring that detects disease earlier, personalized therapy, and reduces the burden of clinic visits and blood sampling.

Beyond wearables, another study tackled the acidic microenvironment that drives invasion [123]. Their tri-anchored methylene-blue pH sensor, encapsulated beneath a Nafion anti-fouling layer and paired with an on-chip Ag/AgCl reference, quantified extracellular pH shifts from 7.4 to 6.4 inside orthotopic tumors with <0.02 pH-unit drift over 72 h. Real-time read-outs correlated with episodes of hypoxia and tumor metabolic fluctuations, offering a direct window into microenvironmental stress and therapeutic response.

**Figure 4:**
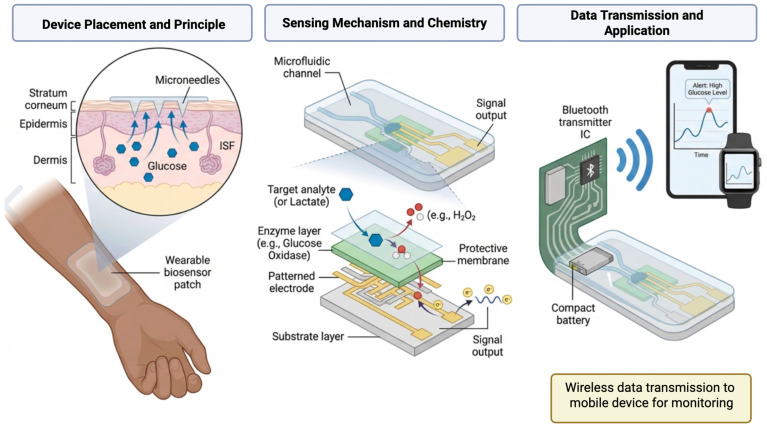
Schematic overview of a wearable electrochemical biosensor for continuous, non-invasive biomarker monitoring. The device is applied as a skin-mounted patch incorporating microneedles that access interstitial fluid beneath the stratum corneum, enabling extraction of target analytes such as glucose or lactate. Within the integrated microfluidic architecture, analytes interact with an enzyme-functionalized sensing layer (e.g., glucose oxidase), producing electrochemical signals through redox reactions at patterned electrodes. The resulting signal is processed and wirelessly transmitted via a compact Bluetooth module to external devices, including smartphones or smartwatches, allowing real-time monitoring and alert generation. This platform highlights the potential of wearable biosensors to replace invasive blood sampling and enable continuous, patient-centric physiological monitoring.

Together, these studies move implantable electrochemistry from proof-of-concept to actionable oncology tools: multiplexed aptamer arrays map dynamic molecular signaling within tumors; bioresorbable films provide short-term surveillance without follow-up surgery; chip-scale implants deliver untethered, high-bandwidth telemetry; and biochemical pH probes track the tumor microenvironment itself. By embedding molecular sense-and-report capability directly within neoplastic tissue, such devices promise earlier relapse alerts, personalized treatment adaptation, and mechanistic insights unattainable with intermittent blood tests or imaging alone. Figure 4 illustrates a mechanism for wearable biosensors.

### 5.3. AI-Integrated Electrochemical Biosensors

Pairing modern electrochemical hardware with machine-learning (ML) analytics transforms the sizable, information-dense waveforms these sensors generate into precise clinical insights. Rather than collapsing a voltammetric or impedance trace to a single peak or resistance value, ML algorithms learn multidimensional patterns within the raw data and output diagnostic probabilities or disease-severity scores in real time.

Researchers fabricated a silver-nanoparticle immune electrode (CHI660E, CH Instruments, Austin, TX, USA) for the breast-cancer proteins CA 15-3 and MUC-1 and collected full cyclic-voltammetry curves for every clinical specimen [124]. They automatically extracted 24 numerical descriptors from each scan—peak currents, integrated charges, derivative features—and trained a random-forest classifier on this feature set. The model distinguished tumors from healthy samples with 98% accuracy. A separate three-layer back-propagation neural network, fed exclusively with CA 15-3 data, correctly stratified histological tumor grade in 76% of cases. Both trained networks were compiled to run on a Raspberry Pi attached to the potentiostat, enabling on-device inference without external computing resources or cloud connectivity and effectively converting the disposable immunosensor into a self-interpreting diagnostic cartridge.

At a deeper level of integration, broadband electrical-impedance spectra (ranging from 100 Hz to 1 MHz) were streamed from a handheld probe into a one-dimensional convolutional neural network [125]. The network learned frequency-dependent impedance signatures characteristic of normal, low-risk, and high-risk oral tissue and returned a malignancy probability within seconds at the chairside. During cross-validated testing, the model achieved an area under the receiver-operating-characteristic curve of 0.91, with 0.97 specificity and 0.74 sensitivity for high-risk lesions; risk-weighted threshold adjustment further improved sensitivity beyond 0.80 with minimal loss of specificity. Because the trained network runs locally on a standard laptop connected to the probe via Bluetooth, the entire system functions as a point-of-care screening tool that delivers near-instant feedback and could reduce unnecessary biopsies.

Ultimately, these studies illustrate how embedding lightweight ML pipelines directly alongside electrochemical transducers elevates the devices from concentration meters to autonomous decision-support systems. By harnessing the complete information embedded in electrochemical waveforms, AI-enhanced sensors enable earlier diagnosis, precise disease staging, and dependable operation in decentralized settings, thereby advancing continuous, patient-focused cancer monitoring. However, translating these advances into clinical workflows remains challenging due to privacy and security requirements of patient data and the need to train clinicians on AI interpretation. Thus, addressing these barriers through standardized frameworks and collaborative efforts between engineers, clinicians, and regulators will be essential to ensure that these platforms are both technically robust and clinically usable.

### 5.4. Microfluidics and Lab-on-a-Chip Platforms

Miniaturized fluidic networks carved into plastics, papers, or 3-D-printed substrates now combine sample preparation, reagent mixing, and electrochemical read-out on a single palm-sized cartridge. Their high surface-to-volume ratios shorten diffusion distances, while integrated valves and mixers automate multi-step assays, allowing clinically relevant cancer biomarkers to be measured from a few microlitres of blood or serum within minutes, outside a central laboratory.

A recent example is the filter-electrochemical microfluidic chip (FEMC) for breast-cancer exosome profiling [126]. Whole blood is first driven through a two-stage membrane stack that enriches vesicles and directs them over four screen-printed electrodes coated with capture antibodies. Zirconium metal–organic frameworks loaded with methylene blue bind phosphate groups on the exosome surface and amplify the current response. The fully enclosed workflow finishes in one hour, achieves a limit of detection of 10^4^ particles mL^−1^, and, by simultaneously quantifying PSMA, EGFR, CD81, and CEA, correctly classified clinical and murine breast-cancer samples into molecular subtypes in a single run.

To demonstrate multiplex protein analysis in a truly disposable format, scientists 3-D-printed a microfluidic immunosensor that routes culture media from 3-D tumor spheroids across nanocomposite electrodes formed from gold nanoparticles and carbon nanotubes [126]. The chip detects carcinoembryonic antigen (CEA) and the proliferation marker Ki-67 in parallel, each with a limit of detection of 0.97 ng mL^−1^, and tracks biomarker knock-down after siRNA treatment—showing how low-cost printed circuitry can support both diagnostic screening and therapy monitoring on the same platform.

Portability and clinical validation were emphasized in the development of a carbon-nanofiber/porous-gold nanoelectrode embedded within a polymethyl-methacrylate microchannel for prostate-specific antigen (PSA) testing [127]. The dynamic hydrogen-bubble template method produced a high-surface-area gold scaffold that, after antibody immobilization, detected PSA down to 5 pg mL^−1^ across a 0.01–50 ng mL^−1^ range. Single-step sample loading and reagent flow gave a total assay time of 21 min, a six-fold reduction versus clinical ELISA, and the device accurately quantified PSA in serum from thirty prostate-cancer patients, confirming its readiness for point-of-care deployment.

Overall, these studies demonstrate how microfluidic lab-on-a-chip engineering can bring sophisticated electrochemical assays to bedside and field settings. By shrinking fluid handling, lowering sample volume, and integrating nanostructured electrodes, the platforms deliver laboratory-grade sensitivity in minutes, support multiplex biomarker panels, and interface readily with smartphones or low-power potentiostats—key attributes for early cancer detection, perioperative surveillance, and routine monitoring in resource-limited environments.

## 6. Challenges and Future Perspectives

Although there has been significant progress in developing cancer biosensors, there are still several key challenges that should be overcome to help progress these technologies into clinical practice.

While many of the discussed biosensors demonstrate exceptional analytical sensitivity—achieving ranges of femtomolar to picomolar detection limits—, only a limited number have advanced to regulatory validation or clinical trial evaluation, highlighting the current gap between laboratory performance and real-world diagnostic implementation. As highlighted across multiple cited studies in this review, many platforms are evaluated primarily in spiked commercial matrices or simple buffer solutions, which are not representative of the environments found in clinical samples. In real patient samples (blood, serum, etc.), non-specific binding of extraneous biomolecules and biofouling of sensor surfaces can produce background signals and degrade sensor performance. Advanced nanomaterial-based sensors that perform well in controlled laboratory buffers also often encounter signal instability, reduce reproducibility, and lose sensitivity when exposed to in vivo-like conditions. Only a minority of published studies examined in this review employed real patient samples or included statistically meaningful clinical cases, making it difficult to assess reproducibility, robustness, and diagnostic metrics—such as sensitivity, specificity, and accuracy—in clinical practice. These gaps highlight the need for more translational validation in order to determine which of the aforementioned biosensing strategies possess valid clinical applicability and which require further optimization before consideration as a clinical tool.

There are also other practical barriers which limit the clinical translation of electrochemical cancer biosensors. For example batch-batch variability in sensor fabrication and differences in assay protocols between laboratories prove to be important challenges. There are also several regulatory approval hurdles as only a few POC electrochemical diagnostics have FDA clearance, and those have been developed through extensive standardized validation in multi-site trials. Lastly improvements are required in both the electrodes and the mediators to improve reproducibility.

To counter these problems, researchers are designing antifouling coatings and robust interface materials that protect the sensing layer without dulling its responsiveness. Hydrophilic nanocomposites, engineered polymer brushes, and other advanced coatings have shown encouraging results in prolonging sensor life and reducing non-specific binding. Alternative recognition strategies, such as molecularly imprinted polymers, are being refined to resist degradation while maintaining high specificity.

Multiplex electrochemical biosensors specifically hold great promise for comprehensive cancer screening, but they are still largely in development. From a regulatory perspective, these multiplex panels require extensive validation for each analyte in the panel. The field must adopt rigorous experimental design and statistical reporting, as assays should be tested in patient matrices and compared side-by-side with gold standards. Clinical implementation will depend on addressing cross-validation, robustness, and standardization issues as outlined above.

Regulatory approval adds another layer of complexity. Before a biosensor can reach patients, it must pass exhaustive validation to prove its accuracy, reliability, and safety while also meeting stringent manufacturing and quality standards. This process demands both technical excellence and careful planning for scale-up, quality control, and post-market monitoring. For smaller teams and startups, the cost and expertise required to navigate these requirements may be challenging. The most successful pathways to approval will likely involve early and ongoing collaboration between engineers, clinicians, and regulatory experts. Multi-center clinical studies and rigorous statistical design are needed for more widespread FDA clearance.

While these challenges are substantial, the opportunities for this technology are equally compelling. The portability and adaptability of electrochemical biosensors make them ideal candidates for future cancer monitoring tailored to each patient and conducted beyond the walls of traditional clinics. The trajectory of electrochemical biosensors points toward a future where continuous, real-time cancer diagnostics are embedded into routine healthcare, allowing for earlier interventions, personalized treatments, and improved patient outcomes.

## 7. Conclusions

Electrochemical cancer biosensors have strong potential to transform oncology diagnostics by enabling rapid, sensitive, and portable detection of disease-specific biomarkers. Their trajectory points toward a convergence of technical innovation, clinical pragmatism, and patient empowerment. By solving the intertwined problems of stability, validation, and usability, the field can shift from producing innovative prototypes to delivering indispensable diagnostic tools. The future of these platforms lies in their seamless integration into clinical workflows, wearable devices, and connected health systems which would enable continuous and personalized monitoring outside of traditional healthcare settings. With sustained innovation and careful translation, electrochemical biosensors can become an important platform for cancer management.

## Figures and Tables

**Table 1:** Summary of primary electrochemical transduction methods.

Electrochemical Transduction Method	Advantage(s)	Limitation(s)	Clinical Practicality
Amperometric	High sensitivity; Versatile integration into devices; Compatible with multiple assay formats	Signal depends on biorecognition element stability; prone to background interference	Adaptable for detecting clinically relevant cancer biomarkers in diagnostic laboratories and point-of-care platforms
Potentiometric	Expanded range of detectable biomarkers; label-free detection of exosomes, miRNAs, and circulating tumor DNA	Lower sensitivity for macromolecules	Well-suited for tumor microenvironment monitoring and integration into FET formats to broaden detectable biomarker ranges
Impedimetric	Label-free, sensitive	Difficulty in miniaturization for point-of-care testing	Capable of detecting clinically relevant cancer biomarkers and has the capacity for integration into point-of-care testing
Voltammetric	Low detection limits; Provides detailed redox profiles; Capacity to detect multiple biomarkers	Requires complex and costly instrumentation	Use in multiplexed assays to detect multiple biomarkers in diagnostics

**Table 2:** Comparison of Diagnostic Methods.

Method	Cost	Detection Time	Sensitivity (LOD)	Specificity
Electrochemical sensor	Low (few $ per test)	Seconds-minutes	Picomolar-femtomolar (pg-fg/mL)	Very High (specific bioreceptor)
ELISA	Moderate ($10–$20/sample)	Hours (2–6 h)	picogram/mL	High (antibody-based)
PCR (qPCR)	High (instrument + $5–$20/sample)	Hours (4–8 h)	Attomolar (few DNA copies)	High (primer-specific)

**Table 3:** Summary of Cancer Biomarkers, Clinical Relevance, and Electrochemical Detection methods.

Cancer Biomarker	Biomarker Type	Proposed Use	Detection Method	Reference
C-reactive protein (CRP)	Protein	-Inflammatory marker associated with cancer prognosis. -Utilized to monitor cancer progression and recurrence. -Aid in diagnosis alongside other markers (e.g., CALLY index).	-CRP-affinity peptide-functionalized label-free electrochemical biosensor -Electrochemical aptasensor -Origami paper-based electrochemical assay -Disposable paper-based electrochemical assay	[39,40,41,42]
Carcinoembryonic antigen (CEA)	Protein	-Monitor recurrence of cancers specifically useful with colorectal cancer.	-Integrated multiplex biosensor assay -Electrochemical paper-based detection -Enzyme-free immunosensor	[43,44,45]
Prostate-specific antigen (PSA)	Protein	-Screening and monitoring prostate cancer.	-Disposable electrochemical aptasensor -Label-free aptasensor -Dual monitor electrochemical biosensor -Glycosylation analysis	[46,47,48,49,50,51,52]
Epidermal growth factor receptor (EGFR)	Genetic	-Identify mutations linked to cancers such as glioblastoma.	-Label free electrochemical biosensor -Capacitive biosensor	[50]
KRAS	Genetic	-Detect oncogenic mutation seen in colorectal, lung, and pancreatic cancer.	-DNA biosensor assay -Nanostructured electrochemical genosensor	[52,53,54]
miRNA	Genetic	-Detect abnormal miRNA expression.	-Microfluidic biochip	[55]
DNA methylation patterns	Genetic	-Detect epigenetic changes such as methylation patterns.	-Polymer Nanobeads and Electrochemical biosensor -Sensing platform based on Ag Nanoparticles	[56,57,58]
Lactate	Metabolic	-Monitor tumor metabolism and aggression.	-Microfluidic organ-on-chip electrochemical system -Dual-sensing system	[59]
Glucose	Metabolic	-Monitor glycolysis activity in tumor cells.	-Microfluidic organ-on-chip electrochemical system -Dual sensing system	[59]

**Table 4:** Summary of Electrochemical Cancer Biomarker Detection Platforms with Analytical Metrics and Validation Parameters.

References	Biomarker/Target	Sensor Type/ Modification	Detection Limit (LOD)	Linear Range	Testing Medium	Interference Study	Real-Sample Validation
Sun et al. [89]	miRNA-155 (breast cancer)	Flexible GFET, defect-free vdW contacts	1.92 fM	10 fM–100 pM	Human serum; human sweat	Not specified	Yes; validated in human serum and sweat samples
Zhou et al. [90]	Carcinoembryonic antigen (CEA)	GFET, antibody-modified (non-covariant)	<100 pg/mL	Not specified	Buffer solutions	Not specified	Not reported
Tao et al. [91]	CEA (Colorectal cancer)	GO nanocomposite, PPI/GO/GCE	0.3 pg/mL	0.1 pg/mL–1000 ng/mL	Buffer/model solutions	Not specified	Not reported
Kalkal et al. [92]	Neuron-specific enolase (NSE, lung cancer)	Amine-N-GQDs/AuNPs fluorescent biosensor	0.09 pg/mL	0.1 pgm/mL–1000 ng/mL	Buffer/model solutions	Not specified	Not reported

## Data Availability

No new data were created or analyzed in this study.
